# Epidemiological changes of acute respiratory infections in children: A single-center experience after COVID-19 lockdown

**DOI:** 10.1371/journal.pone.0300877

**Published:** 2024-04-05

**Authors:** Indrė Stacevičienė, Inga Ivaškevičienė, Sigita Burokienė, Aušra Steponavičienė, Daiva Vaičiūnienė, Gabrielė Tarutytė, Augustina Jankauskienė

**Affiliations:** Faculty of Medicine, Vilnius University, Vilnius, Lithuania; University of Ilorin, NIGERIA

## Abstract

**Background:**

Since the start of the COVID-19 pandemic, the epidemiology of acute respiratory infections (ARIs) has continually changed, making it difficult to predict. Our study aimed to evaluate epidemiological changes and clinical outcomes of ARIs in pediatric patients in the post-lockdown period.

**Methods:**

A single-center retrospective cross-sectional study was performed in one of the largest pediatric emergency departments in Lithuania during two cold seasons–from October 1, 2021, to April 30, 2022 (Season I) and in the same period in 2022–2023 (Season II). Patients under 18 years of age who had been tested for COVID-19 were enrolled in the study. Additional data about other respiratory pathogens in the study group (specifically influenza A/B, respiratory syncytial virus (RSV) and group A *Streptococcus* (GAS)), were included.

**Results:**

During both seasons of our study, 19,366 children were screened for COVID-19. Positive tests for COVID-19 decreased from 14.5% in Season I to 5.9% in Season II, while at the same time, the rates of other infections increased significantly: influenza from 17.5% to 27.1%, RSV from 8.8% to 27.6%, and GAS from 8.4% to 44%, respectively. In Season II, COVID-19 infection presented in fewer admissions to pediatric intensive care (0.8% vs. 3.7%, p<0.01) and there were no deaths, while influenza presented in a higher proportion of hospitalizations (10.5% vs. 6.1%, p<0.01) and there was one death. The proportion of RSV hospitalizations also increased in Season II (34.6% vs. 44.0%, p<0.01).

**Conclusions:**

The early post-lockdown period saw a decline of COVID-19 and re-emergence of influenza, RSV and GAS infections in children. In Season II, COVID-19 cases became milder contrary to influenza. RSV infection contributed significantly to hospitalizations for respiratory infections in children in both seasons, particularly in Season II. Coinfections were not associated with a more severe course of the disease.

## Introduction

Acute respiratory infections (ARIs) are the leading infectious disease among children globally and are responsible for a significant proportion of pediatric hospitalizations and deaths [1, 2]. From the time the SARS-CoV-2 virus emerged in 2019 and in the COVID-19 pandemic that followed, the epidemiology of ARIs has continually changed, making it difficult to predict. Although there was an initial decrease in overall viral infections during the first and second lockdown periods, they were followed by significant increases in respiratory infections after preventive measures were relaxed [3, 4].

During Season I (2021–2022), countries reported changes in the distribution of ARIs and different incidence peaks than had trended in the years before COVID-19 [5]. These differences continued during Season II (2022–2023) [3, 4]. More time and data are needed to see the complete picture.

In Lithuania, the second nationwide lockdown ended on July 1, 2021, after which infection control measures were progressively phased out, leading to their complete cessation. The aim of this study was to evaluate the epidemiological changes and clinical outcomes of ARIs in pediatric patients in the post-lockdown period (Season I and Season II) in one of the largest pediatric emergency departments in Lithuania.

## Materials and methods

A single-center retrospective cross-sectional study was performed during two cold seasons, from October 1, 2021 to April 30, 2022 (Season I) and in the same period in 2022–2023 (Season II) at the Vilnius University Hospital Santaros Klinikos. This study was approved by the Vilnius Regional Biomedical Research Ethics Committee (No. 2020/8-1269-737) and conducted in accordance with the principles of the World Medical Association Helsinki Declaration as well as local law.

Since this research was a continuation of previously performed studies [6, 7], the inclusion criteria were as follows: patients under 18 years of age tested for COVID-19 in the pediatric emergency department (PED) during the time frame of the study. The analysis was performed from the perspective of the laboratory records. The clinical factors that determined whether a child was tested for other pathogens were not available and thus not considered. However, if these patients were also tested for other respiratory pathogens, specifically influenza A/B, respiratory syncytial virus (RSV) and group A *Streptococcus* (GAS), those data were included and analyzed. Double entries were excluded.

During Season I, nasopharyngeal swabs were tested using real-time reverse-transcriptase polymerase chain reaction (RT-PCR) tests for SARS-CoV-2, influenza A/B viruses, and RSV using the Xpert Xpress CoV-2/Flu/RSV plus (Cepheid) with 100% specificity and 100% sensitivity. During Season II, depending on the availability of the tests, patients were tested with the same RT-PCR tests or Flowflex SARS-CoV-2 & Influenza A/B Ag Combo Rapid Test (Acon, Biotech) with >99% specificity and >97% sensitivity. During both seasons, GAS antigens from throat swabs were detected by QUIDEL’s Strep A test, using immunofluorescence technology with 93.7% specificity and 94.4% sensitivity.

Basic characteristics (age, gender, date of investigation) and outcome data (hospitalized/discharged from PED, duration of hospitalization, admissions to the pediatric intensive care unit (PICU), recovered/deceased) were obtained by biostatisticians from patient electronic medical records. For any deaths, data of underlying diseases were also gathered by the International Statistical Classification of Diseases and Related Health Problems, 10^th^ revision (ICD-10). An additional analysis was conducted of coinfections (COVID-19 & Influenza A/B; COVID-19 & RSV; COVID-19 & GAS; Influenza A/B & RSV; Influenza A/B & GAS; RSV & GAS) and resulting outcomes. The authors received an encrypted database (12 July, 2023) and therefore did not have access to information that could identify individual participants during or after data collection. The Vilnius Regional Biomedical Research Ethics Committee authorized the use of data without the need for informed consent, since the data were obtained retrospectively and anonymously.

Statistical analyses were performed with R software (version 4.2.2, R Foundation for Statistical Computing, Vienna, Austria). Categorical data were presented as frequencies and percentages and analyzed using Pearson’s chi-square or Fisher’s exact test where appropriate, to ascertain differences between seasons. These statistical methods were also used to determine the relation between co-infections, hospitalization and admission to the PICU. We used the chi-square goodness of fit test to perceive statistically significant differences in seasons by month for different infections. For continuous data, medians/interquartile range (IQR) were calculated, and the Wilcoxon rank sum test was used for age comparison. A p < 0.05 was considered as statistically significant.

## Results

A total of 19,366 children were screened for SARS-CoV-2 in both seasons: 12,741 in Season I and 6,625 in Season II. COVID-19 was more common in Season I, with significant increases of influenza, RSV and GAS infections in Season II (Table 1).

**Table 1:** Distribution of tests and positive results in Seasons I and II.

Characteristics	Season I (2021–2022)			Season II (2022–2023)			*p*	TOTAL (Season I and II)	
	Tests no	Positive tests no	Positive tests %	Tests no	Positive tests no	Positive tests %		Positive tests no	Positive tests %
COVID-19 RT-PCR^a^ test	12741	1847	14.5	2646	158	6.0	p = 0.000	2005	13.0
COVID-19 rapid test	0	-	-	4000	238	6.0	-	238	6.0
**COVID-19 TOTAL**	**12741**	**1847**	**14.5**	**6625**	**393**	**5.9**	**p = 0.000**	**2240**	**11.6**
Influenza A/B RT-PCR^a^ test	5015	878	17.5	1954	631	32.3	p = 0.000	1509	21.7
Influenza A/B rapid test	0	-	-	3166	757	23.9	-	757	23.9
**Influenza TOTAL**	**5015**	**878**	**17.5**	**5110**	**1386**	**27.1**	**p = 0.000**	**2264**	**22.4**
**Respiratory syncytial virus**	**4876**	**431**	**8.8**	**2138**	**590**	**27.6**	**p = 0.000**	**1021**	**14.6**
**Group A *Streptococcus***	**320**	**27**	**8.4**	**1674**	**737**	**44.0**	**p = 0.000**	**764**	**38.3**

^a^RT-PCR = real-time reverse-transcriptase polymerase chain reaction.

### Gender and age differences

**Fig 1:**
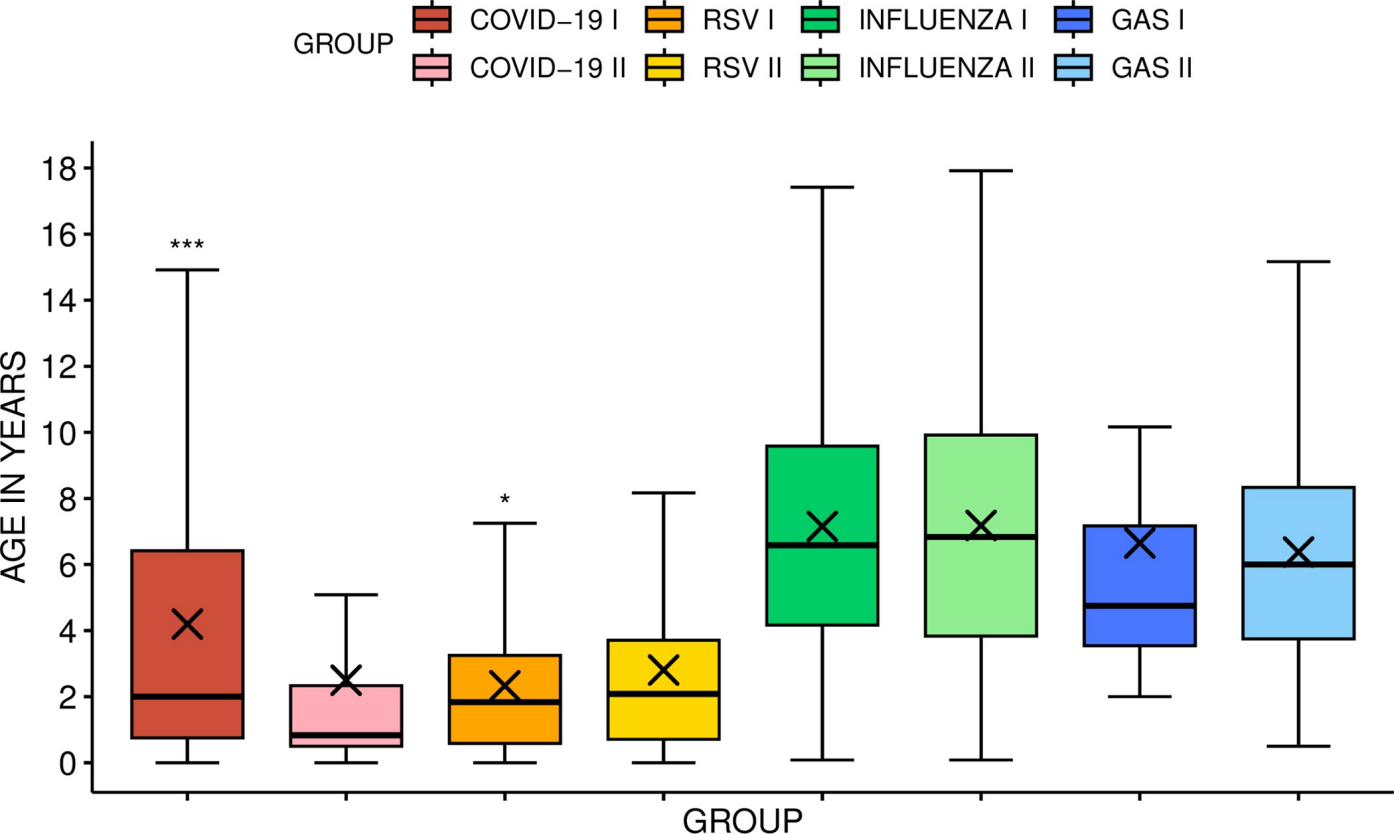
Comparison of age distribution during Seasons I and II. The figure includes whiskers, quartiles, median and sample mean (the cross). * p<0.05; *** p<0.001. COVID-19 = Coronavirus disease 2019; RSV = respiratory syncytial virus; GAS = group A *Streptococcus*; I = Season I (2021–2022); II = Season II (2022–2023).

Distribution by gender was similar among children in both seasons, with a significant difference in age (Fig 1). The majority of patients with either COVID-19 or RSV were between 0–3 years old (64.2% of COVID-19 cases in Season I and 81.9% in Season II; RSV 83.5% and 77.1%, respectively). More infants (<1 yr) were among the COVID-19 patients in Season II compared to Season I (54.2% vs. 31.3%, p = 0.000, respectively) with no significant difference among RSV patients (30.3% vs. 32.3%, respectively).

A slight majority of patients with influenza and GAS infections were 4–9 years old (52.7% of influenza cases in Season I and 49% in Season II; GAS: 40.7% and 57.7%, respectively). In Season I, GAS infection was not detected in patients under 2 years old; in Season II, GAS infection was found in 6.9% (n = 51) of this age group. There were no significant age differences among influenza patients between the two seasons.

### Seasonal differences

**Fig 2:**
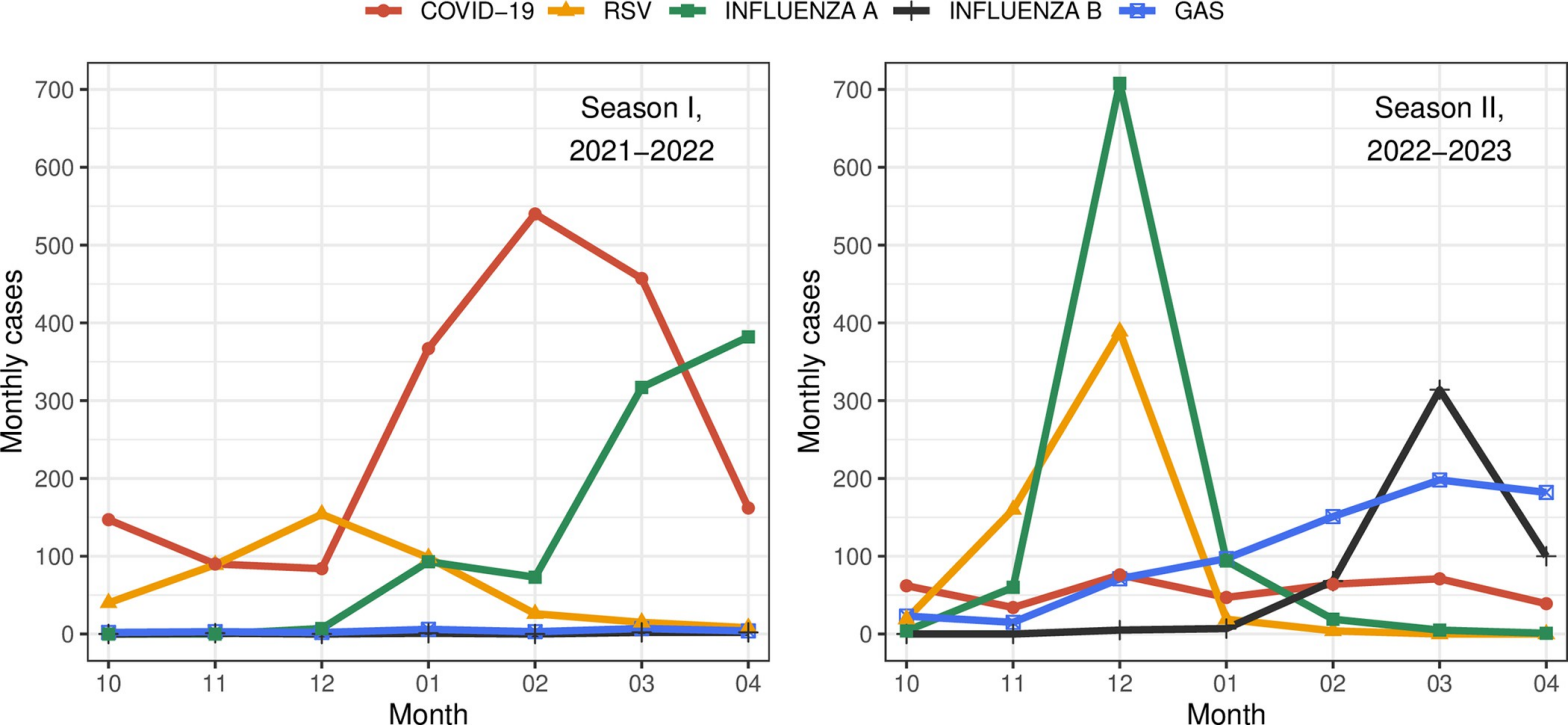
Monthly distribution of COVID-19, influenza A/B, RSV and GAS cases during Seasons I and II. COVID-19 = Coronavirus disease 2019; RSV = respiratory syncytial virus; GAS = group A *Streptococcus*. Significant differences by month (* p<0.05; ** p<0.01; *** p<0.001): COVID-19 (10, 11, 12, 01, 02, 03) ***; RSV (10, 11, 12, 01, 02, 03, 04) ***; Influenza A (10, 11, 12, 02, 03, 04) ***; Influenza B (11, 12, 01, 02, 03, 04) ***; GAS (10, 11, 01, 02, 03, 04) ***, (12)**.

Most COVID-19 cases in Season I were diagnosed in January-March, peaking in February; in Season II there was no peak, the curve being nearly flat (Fig 2). In Season I there was only one wave of influenza A, peaking in March-April. There were two waves of influenza in Season II, the first caused by influenza A, peaking in December, and the second caused by influenza B in February-April, peaking in March. RSV infection was predominant in December in both seasons. Cases of GAS infection slightly increased each month from December 2022 through the end of Season II.

### Coinfections

**Fig 3:**
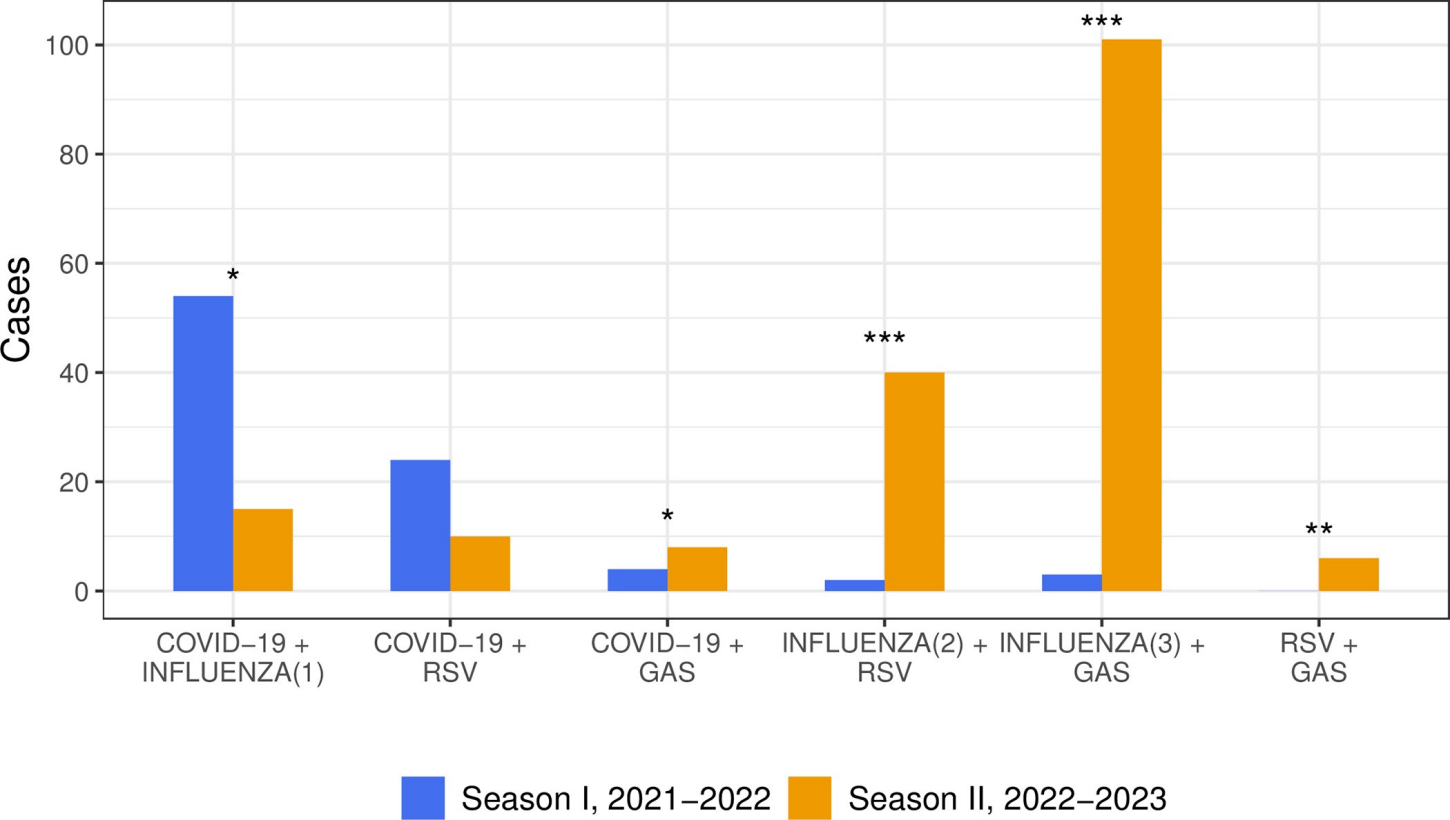
Distribution of coinfections during Seasons I and II. * p<0.05; ** p<0.01; *** p<0.001. COVID-19 = Coronavirus disease 2019; RSV = respiratory syncytial virus; GAS = group A *Streptococcus*. ^1^ In Season I there were 54 COVID-19 coinfections with influenza A and no coinfections with influenza B; during Season II there were 12 COVID-19 coinfections with influenza A and 3 with influenza B; ^2^ In both seasons RSV coinfections were only with influenza A; ^3^ In Season I there were 3 GAS coinfections with influenza A and no coinfections with influenza B; during Season II there were 40 GAS coinfections with influenza A and 61 with influenza B.

More coinfections were detected during Season II than Season I (180 vs. 87, respectively). Coinfections of COVID-19 with influenza A or RSV were predominant in Season I, whereas coinfections of influenza A with RSV and coinfections of influenza A/B with GAS dominated in Season II (Fig 3).

### Outcome data

The proportion of hospitalizations for COVID-19, influenza and RSV patients was significantly higher during Season II (Table 2). The duration of hospitalization was similar among all tested infections during both seasons. Admission rates to PICU were significantly higher among patients with COVID-19 during Season I compared to Season II, with no significant differences among other infections. Coinfection outcome data (in terms of hospitalization, admission to PICU) did not display meaningful differences.

**Table 2:** Outcome data of single infections of COVID-19, influenza, RSV, and GAS, and their coinfections in Seasons I and II.

Charcteristics	Total no.		Median age, years (IQR)		Hospitalization no. (%)		Average duration of hospitalization—days–median (IQR^e^)		Admission to PICU^f^ no. (%)		Death no. (%)	
Season	I	II	I	II	I	II	I	II	I	II	I	II
**COVID-19**	1765	361	1.9 (0.8–6.2)	0.75 (0.5–1.7)	417 (23.6)**	110 (30.5)**	3.0 (2.0–4.0)	3.0 (2.0–3.3)	66 (3.7)**	3 (0.8)**	2 (0.1)	0
**Influenza**	819	1231	6.6 (4.2–9.6)	6.8 (3.8–10.2)	50 (6.1)**^a^	129 (10.5)**^b^	3.0 (2.0–4.0)	3.0 (2.0–4.0)	5 (0.6)	6 (0.5)	0	1 (0.1)
**RSV** ^**c**^	405	534	1.8 (0.6–3.3)	2.0 (0.7–3.5)	140 (34.6)**	235 (44.0)**	4.0 (3.0–5.8)	4.0 (3.0–5.0)	12 (3.0)	15 (2.8)	0	0
**GAS** ^d^	20	623	4.8 (3.6–12.1)	5.9 (3.7–8.3)	4 (20.0)	116 (18.6)	3.0 (2.0–4.0)	3.0 (3.0–5.0)	0	7 (1.1)	0	0
**Co-infections**												
**COVID-19 + influenza**	54	15	6.5 (4.2–9.5)	8.6 (3.1–10.0)	2 (3.7)	3 (20.0)	2.5 (2.0–2.5)	6.0 (2.0–6.0)	1 (1.9)	0	0	0
**COVID-19 + RSV** ^**c**^	24	10	1.2 (0.8–2.6)	2.4 (0.4–4.4)	5 (20.8)	3 (30.0)	4.0 (2.5–4.5)	3.0 (1.0–3.0)	0	0	0	0
**COVID-19 + GAS** ^d^	4	8	4.6 (2.7–7.4)	4.7 (2.7–6.6)	0	2 (25.0)	-	5.0 (3.0–5.0)	0	0	0	0
**Influenza + RSV** ^**c**^	2	40	5.9 (4.2–5.9)	4.0 (2.1–6.8)	0	7 (17.5)	-	4.0 (2.0–5.0)	0	1 (2.5)	0	0
**Influenza + GAS** ^d^	3	101	4.7 (3.4–4.7)	7.3 (4.4–9.3)	1 (33.3)	14 (13.9)	2.0 (2.0–2.0)	3.0 (2.8–6.3)	0	0	0	0
**RSV**^**c**^ **+ GAS**^d^	0	6	-	5.3 (3.3–6.5)	0	1 (16.7)	-	5.0	0	0	0	0

** p<0.01

^a^ during Season I 47/813 of influenza A and 3/7 of influenza B cases were hospitalized

^b^ during Season II 79/799 of influenza A and 50/432 of influenza B cases

^c^ RSV = respiratory syncytial virus

^d^ GAS = group A *Streptococcus*

^e^ IQR = the interquartile range

^f^ PICU = the pediatric intensive care unit.

There were two deaths among COVID-19 patients during Season I, a 5-year-old boy and a 10-year-old girl with an underlying oncohematological disease. There was one death in Season II, of 6-year-old boy with influenza B and no underlying diseases. None of these patients had a coinfection with the other described respiratory pathogens.

## Discussion

During the COVID-19 pandemic, circulation of respiratory pathogens decreased dramatically as infection control measures were taken to diminish the burden on health care systems. Although the pandemic as a global health emergency lasted more than 3 years (until the beginning of May 2023), infection control measures were released over time [8], resulting in the return of the respiratory infection burden [3, 4]. Our study presents epidemiological changes of ARIs in children during the two different pandemic seasons that followed strict lockdowns.

We tested over 19,000 children of different age groups for SARS-CoV-2 at our tertiary hospital. COVID-19 dominated during Season I, reaching a peak in February, while in Season II the number of cases dropped sharply (with a 4.7-fold reduction) with the incidence curve flattening and peaks no longer registering. These data reflect the national COVID-19 statistics for all age groups showing an even more significant (12-fold) reduction in total cases compared to the previous season [9]. A general downward trend was also seen in other European countries [10]. Beginning with Season II, the epidemiological picture of COVID-19 infection in Europe has been characterized by lower periodic waves, approximately every 2–3 months [10].

Data from our study shows a higher proportion of hospitalized COVID-19 patients during the second season. This may be related to the higher rate of SARS-CoV-2 cases among infants who, as the youngest, require more care and assistance. It is important to emphasize that the duration of hospitalization did not differ between the two seasons. The absolute number of COVID-19 hospitalizations and the proportion of admissions to PICU were lower, with no deaths among SARS-CoV-2 patients during Season II. This most likely reflects the circulation of milder forms of the disease. This trend is also reflected in Lithuania nationally, in which there was a significant reduction in deaths from COVID-19, from 4,032 in Season I to 351 in Season II across all age groups [9].

The influenza virus was the second-most prominent respiratory pathogen found in over 10,000 children who were tested, with a significant 1.6-fold increase of influenza cases detected during Season II. According to the National Centre for Public Health (NCPH) of Lithuania, during the 2020–2021 season, only 302 laboratory confirmed influenza cases were recorded in the country. As soon as COVID-19 infection control measures were relaxed, influenza cases re-emerged: 6,032 cases in Season I followed by a 9-fold increase (n = 54,808) in the next cold season [9].

In our study, only a single late wave of influenza A was observed in Season I, reaching its peak in March-April. Influenza B cases were sporadic during this time period. The European Centre for Disease Prevention and Control (ECDC) data show similar results: an unprecedented late onset of an influenza A wave in many European countries at the same time, 2021–2022 [11]. This late onset may be due to preventive measures implemented during the winter period leading to late activity when measures were released. The following influenza season marked the return of influenza virus activity at almost pre-pandemic levels in European countries with an earlier peak compared to the four previous seasons [12]. Our findings contribute to these data, showing the first wave of influenza caused by influenza A with a peak in December, followed by the second wave caused by influenza B in February-April.

The death caused by influenza B and the 2.6-fold higher proportion of hospitalizations of influenza patients during Season II may be linked to a more severe course of the disease. Nevertheless, the duration of hospitalizations and proportion of admissions to PICU did not differ significantly. Data from the NCPH of Lithuania provides evidence for disease severity, with a 4-fold higher hospitalization rate, increased admissions to the PICU, and an additional death among children in 2022–2023 [9]. During this time, the vaccination coverage rate among children was very low. Only about 10% (n = 286,470) of the total population in Lithuania was vaccinated against influenza, with children comprising only 3% (n = 9,019) of the vaccinated population [9, 13]. Additionally, the low circulation of influenza in the first two COVID-19 pandemic seasons left the population susceptible to influenza. These factors likely contributed to the high rate of influenza-associated pediatric hospitalizations, not only in our study, but throughout the country. According to data in the USA, the 2022–2023 influenza season was classified as having high severity among children and adolescents. During this time period, rates of influenza-associated medical visits and hospitalizations were higher than during most previous seasons for children and adolescents aged <18 years [14]. We did not observe a significant impact by the different influenza types on the severity of disease. We did observe that influenza type A was the dominant type among hospitalized patients in our study, consistent with other European countries [12].

RSV, a leading cause of acute lower respiratory tract infections in infants and young children [15] also affected most of the youngest children (0–3 years) in our study. In Season II proportionately more positive tests were obtained, although in absolute numbers, the observed difference was not as great: from 8.8% to 27.6% and from 431 to 590, respectively. This may be explained by a more appropriate selection of target groups for RSV testing during Season II.

RSV infection was predominant in December in both seasons. However, the sudden increase in RSV cases and higher peak and proportion of hospitalizations in Season II added pressure on the health care system. In addition, the peak of RSV cases coincided with the peak of influenza A cases, resulting in an increase of influenza A/RSV coinfections. Overall, the trends of RSV epidemiological changes are similar to those of influenza: implementation of COVID-19 quarantine interventions in many countries, including Lithuania, resulted in a strong reduction of RSV activity in 2020–2021 followed by a rapid return of RSV cases to pre-pandemic levels in the post-lockdown period (Season I and Season II) [9, 16, 17]. Of particular importance is the inadequate surveillance of RSV infection in Lithuania. Since only partial information is maintained by the NCPH (RSV cases are not routinely collected), the full burden of Lithuania’s RSV infection rate is not known. Our study adds significantly to the data on RSV infection among children, the most vulnerable group.

In Season II the waves of influenza A and RSV were replaced by a gradual increase of GAS cases, unlike the previous season. The peak of influenza B coincided with a peak of GAS infection, resulting in high number of coinfections of these two infections in Season II. Similar to influenza, GAS affected primarily children of 4–9 years. During Season II, GAS was also found among the youngest children (<2 yrs), not typically characteristic for this age group [18]. According to Epic Research, in February 2023, streptococcal tonsillitis increased to a rate nearly 30% greater than the previous peak seen in February 2017. Similar to our study, the most affected group was children 4–9 years old with an increase of GAS cases among the youngest [19]. We have not seen significant differences of GAS outcomes between the two seasons. Coinfection with influenza did not have a notable impact on the course of the disease since there were no related admissions to PICU or deaths. However, this data should be evaluated cautiously because of the comparison of very distinct groups and the small number of cases.

The number of diagnostic tests performed varied between seasons: COVID-19 and RSV testing decreased by almost half over the time periods, influenza testing remained stable, and testing for GAS increased by several times. These differences can be attributed to a change in testing policy and declining demand for testing due to the changing and decreasing incidence of certain infections. As more sensitive, less expensive, and easier to perform rapid antigen tests became available, they were often used to test our patients for SARS-CoV-2 and influenza during Season II after testing policies had relaxed. The use of different tests to diagnose COVID-19 or influenza could be considered a limitation of this study. However, knowing that rapid antigen tests have high specificity and sensitivity, we believe this does not significantly influence the final results. Although the percentage of influenza-positive PCR and rapid tests was slightly different, SARS-CoV-2 detection was the same. Another limitation is that we evaluated SARS-CoV-2, influenza, RSV and GAS activities only, while other human respiratory pathogens were not considered. The number of deaths during the study period was low, but given that the data were collected from only one center, this should be interpreted with caution.

The strength of our study is the high number of children tested for clinically important respiratory pathogens. As these data have been collected in one of the largest pediatric tertiary-level centers in the Baltic States, it adds considerably to our understanding of childhood ARI trends in the post-lockdown period. This knowledge can improve health care strategies and support advocacy for preventive measures, such as vaccination.

## Conclusions

The early post-lockdown period saw a decline of COVID-19 and re-emergence of influenza, RSV, and GAS infections in children. COVID-19 cases became milder with lower admissions to PICU and no deaths, unlike influenza, which presented in a higher proportion of hospitalizations and one death. RSV infection contributed significantly to hospitalizations for respiratory infections in children, especially among the youngest, in both seasons, particularly in Season II. These data evidence the need for more complete monitoring of RSV infection, as is done with COVID-19 and influenza. Coinfections were not associated with a more severe course of the disease.
